# Predicting prognosis according to preoperative chemotherapy response in patients with locally advanced lower rectal cancer

**DOI:** 10.1186/s12885-019-6424-4

**Published:** 2019-12-16

**Authors:** Takuya Shiraishi, Takeshi Sasaki, Koji Ikeda, Yuichiro Tsukada, Yuji Nishizawa, Masaaki Ito

**Affiliations:** Department of Colorectal Surgery, National Cancer Center Hospital East, 6-5-1 Kashiwanoha, Kashiwa, Chiba 277-8577 Japan

**Keywords:** Extramural venous invasion, Neoadjuvant chemotherapy, Locally advanced lower rectal cancer, Tumor volume reduction

## Abstract

**Background:**

Neoadjuvant chemoradiotherapy is regarded as the standard of treatment for locally advanced lower rectal cancer, although some of these cases are systemic, and distant control may be inadequate. Neoadjuvant chemotherapy could compensate for such shortcomings, potentially yielding better survival outcomes. We aimed to stratify patients into prognostic groups on the basis of preoperative factors, including response to neoadjuvant chemotherapy.

**Methods:**

We retrospectively analyzed patients with locally advanced lower rectal adenocarcinoma (clinical stage II/III with high-risk features of distant metastasis) who were treated with neoadjuvant chemotherapy (without radiotherapy) followed by curative resection between 2010 and 2017. Reduction in tumor volume (before vs. after neoadjuvant chemotherapy) was measured using magnetic resonance imaging, and a reduction above 60% was defined as a good response. Recurrence and overall survival were evaluated.

**Results:**

The cohort comprised 102 patients. Good response to neoadjuvant chemotherapy was associated with better 5-year recurrence-free survival (good responders: 81.1%, poor responders: 49.0%; *p* = 0.001) and 5-year overall survival (good responders: 94.9%, poor responders: 80.6%; *p* = 0.06). In a multivariate analysis, extramural venous invasion on magnetic resonance imaging after neoadjuvant chemotherapy and a tumor volume reduction rate < 60 were found to be significantly and independently associated with worse recurrence-free survival (hazard ratio: 2.74, 95% confidence interval: 1.36–5.50, *p* = 0.005 and hazard ratio: 3.48, 95% confidence interval: 1.57–7.72, *p* = 0.002, respectively). Good responders without extramural venous invasion had the best 5-year recurrence-free and overall survival (89.0 and 93.8%, respectively). Poor responders with extramural venous invasion had the worst 5-year recurrence-free and overall survival (21.4 and 50.0%, respectively).

**Conclusions:**

Reductions in tumor volume after neoadjuvant chemotherapy were associated with a better prognosis in patients with locally advanced lower rectal cancer. Extramural venous invasion was a preoperative prognostic factor.

## Background

The prognosis of locally advanced lower rectal cancer (LALRC) might be improved by individualizing treatment. There are two fundamental aspects to the treatment of rectal cancer: interventions to control local disease, such as surgery and radiotherapy, and interventions to control systemic disease, such as chemotherapy and immunotherapy. The standard treatment for LALRC is neoadjuvant chemoradiotherapy (NACRT), followed by total mesorectal excision (TME) [1, 2]. This combination is generally thought to be essential and is performed for almost all patients. Systemic adjuvant chemotherapy is provided after tumor resection, and neoadjuvant chemotherapy (NAC) is optional.

Although fluorouracil-based adjuvant chemotherapy significantly improves overall survival (OS) and recurrence-free survival (RFS), nearly 30% of eligible patients do not receive adjuvant chemotherapy because of their postoperative status [1, 2]. Moreover, in standard treatment, NACRT and surgery delay the start of systemic therapy by approximately 6 months. Approximately 30% of LALRCs are systemic diseases at high risk for distant metastasis, and more distant metastases may occur in patients suspected of having extraluminal lesions, including patients with lymph node metastasis and/or very low tumor location [3–6]. NAC could compensate for such shortcomings, potentially yielding better survival outcomes. However, only few studies have reported the efficacy of NAC for LALRC without NACRT [7, 8].

NACRT strongly improves local control in patients with LALRC progression [6, 9, 10]. The essential role of NACRT derives from its ability to boost local control. However, some cases of LALRC require systemic therapy like NAC, rather than local treatments like NACRT or even surgery. NAC may control distant metastasis and should therefore be considered equally important. However, it is difficult to accurately identify the malignancy grade of LALRC before the start of local treatment.

We considered that the response of LALRC to NAC might predict the emergence of distant recurrence postoperatively. Therefore, we analyzed patients who underwent NAC for LALRC without receiving NACRT to investigate the associations between the effects of NAC and long-term postoperative outcomes. This study aimed to stratify patients with LALRC into prognostic groups based on preoperative information, including response to NAC. Prognosis was compared between patients who showed good and poor responses after chemotherapy. Further, we aimed to identify preoperative prognostic factors that could be obtained before surgery.

## Methods

### Patients and study design

We retrospectively analyzed all patients with locally advanced lower rectal adenocarcinoma (clinical stage II/III with high-risk features of distant metastasis) who received NAC followed by curative resection at the National Cancer Center Hospital East, Japan, between January 2010 and February 2017. During this period, we selected therapeutic strategies involving NAC (instead of NACRT) for patients with LALRC who were thought to be at high risk of distant recurrence. Japanese Society for Cancer of the Colon and Rectum guidelines recommend surgery first followed by adjuvant treatment [11], however more and more NACRT for patients with LALRC is used to decrease local recurrence rate. At this period, we were exploring NAC for the purpose of controlling distant recurrence related to patient survival rather than CRT for local control. The selection criteria were as follows: (1) high risk of distant metastasis (i.e., suspicion of an extramural cancer lesion including multiple lymph node swelling (≥6 mm in the short axis) and of lymph node metastasis (≥6 mm in short axis) in the pelvic sidewall area) and (2) resectable primary lesion with an estimated clinical circumferential resection margin (CRM) of > 2 mm on post-chemotherapy magnetic resonance imaging (MRI) via TME or wider resection, including adjacent organs or nerve tissue and muscles. All patients gave informed consent for strategies that were not standard. From this initial population, all of the patients with primary elective surgery were identified. Those with no MRI data, intolerance to NAC, or with combined chemotherapy with molecular targeted drugs were excluded.

Data on the following clinicodemographic characteristics were extracted from medical and operation reports: age, sex, body mass index, anal verge (AV) distance, carcinoembryonic antigen level, clinical TNM classifications, clinical CRM, extramural venous invasion based on MRI before and after neoadjuvant chemotherapy (mrEMVI and ymrEMVI, respectively), surgical procedure, operation type, pathological TNM classifications, histological type, pathological CRM, distal margin, and adjuvant chemotherapy. The patients were followed until September 2018. This study was approved by our institutional review board (National Cancer Center Hospital Approval no. 2017–349).

### Clinical TNM classifications, clinical CRM, mrEMVI, ymrEMVI, and TVRR

**Fig. 1:**
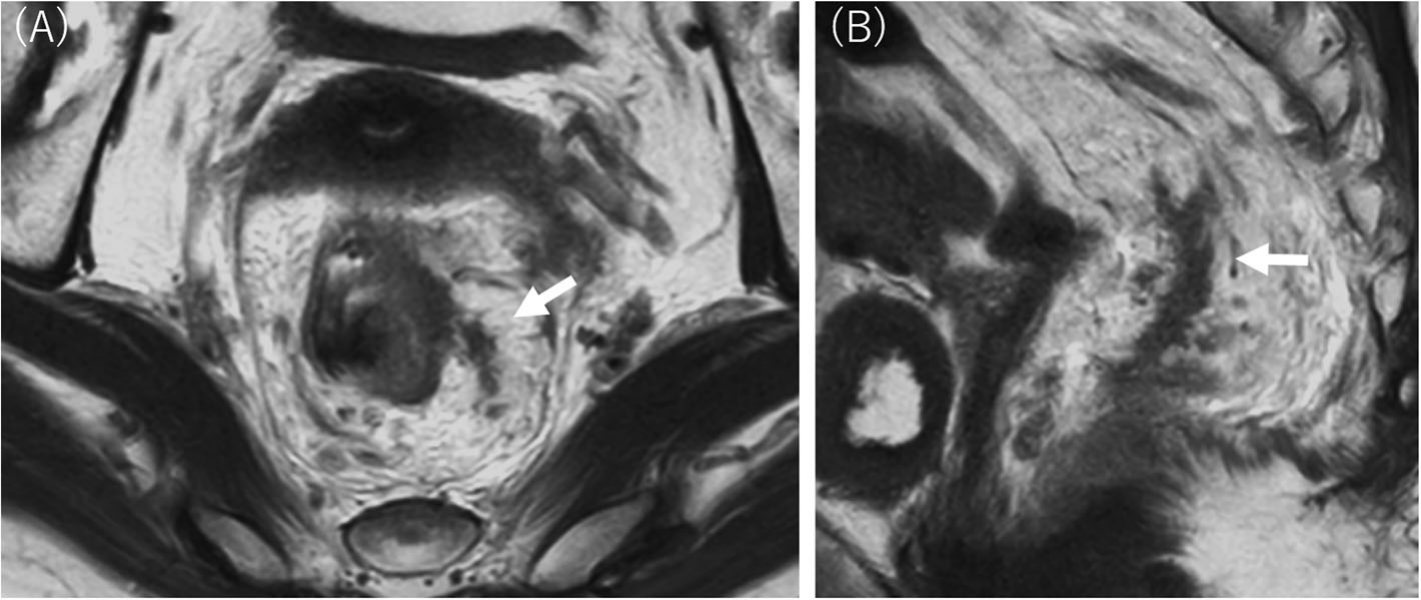
Axial (**a**) and sagittal (**b**) T2-weighted magnetic resonance images. The tumor signal extended into the vascularis outside the muscularis propria of the bowel wall (arrows). This case was scored as a 4

Pretreatment clinical TNM classifications were assessed based on computed tomography (CT) and MRI findings. The tumor location was determined via endoscopy and barium enema. Clinical CRM was measured via MRI before NAC, and clinical CRM positivity was defined as a ≤ 2-mm margin between adjacent organs or nerve tissue, muscles, and the deepest part of the primary lesion. EMVI status was evaluated according to the 5-scale EMVI scoring system [5] and recorded as positive (scores 3 and 4) (Fig. 1) or negative (scores 0, 1, and 2). EMVI status was evaluated before and after neoadjuvant chemotherapy (mrEMVI and ymrEMVI, respectively). The tumor volume reduction rate (TVRR) was also calculated as described previously [12]. The length and width of the tumor were measured on the axial slice of the maximum dimension, and the maximum height was measured on a sagittal slice. Tumor volume was estimated by multiplying tumor length, width, and height. The TVRR was defined as 100 × [(volume baseline − volume post NAC)/volume baseline]. We classified the patients into two groups according to previously established criteria: good responders (those having a TVRR ≥60) and poor responders (those having a TVRR < 60) [13, 14]. The sizes of primary lesions and the presence of mrEMVI and ymrEMVI were evaluated by two experienced colorectal surgeons, and incongruent results were reviewed and finalized by consensus.

### Preoperative chemotherapy, surgery, and postoperative chemotherapy

Patients were generally treated with FOLFOX (folinic acid, fluorouracil, and oxaliplatin), although CAPOX (capecitabine and oxaliplatin) was also administered. All NAC regimens were based on fluorouracil and oxaliplatin. Each physician determined the regimen that the patient received. In general, FOLFOX and CAPOX were administered as 6- and 4-cycle regimens, respectively.

The surgical procedures consisted of low anterior resection, intersphincteric resection (ISR), and abdominoperineal resection, which were performed via the conventional open method or laparoscopic surgery. Laparoscopic procedures began to be used for LALRC in 2012 and gradually became more common thereafter. All of the procedures included lymphadenectomy using the standard TME technique. All patients underwent lateral pelvic lymph node dissection (LPLND). LPLNDs for internal iliac and obturator lesions were performed as described previously [15].

Postoperative adjuvant chemotherapy was generally administered for at least 3 months.

### Statistical analysis

Categorical variables are reported as frequencies (percent) and were analyzed using the Chi-square test or Fisher’s exact test. Quantitative variables are reported as median (range). Survival rates, such as RFS and OS, were estimated using the Kaplan-Meier method. RFS was defined as the period from the date of operation to any recurrence. OS was defined as the time between surgery and death from any cause. Survival differences were assessed using the log-rank test. Variables with *p* < 0.05 on univariate survival analyses were included in multivariate survival analyses, which were performed using the Cox proportional hazards model. Multivariate analyses were used to identify independent predictors of RFS and OS before surgery. Results of the multivariate analyses are reported as hazard ratios (HR) and 95% confidence interval (95% CI). All statistical analyses were performed using SPSS 22.0 (SPSS Inc., Chicago, IL, USA), and *p* < 0.05 was considered statistically significant.

## Results

### Patient characteristics

We treated 450 patients with primary LALRC located below the peritoneal reflection. Of these patients, 120 patients received NAC without NACRT. We excluded 13 patients who did not undergo MRI, 2 patients who underwent only one course of NAC, and 3 patients who received combined chemotherapy with molecular targeted drugs. The final study population consisted of 102 patients who underwent curative surgery after NAC.

The characteristics of the 102 patients are summarized in Table 1. Neoadjuvant regimens included FOLFOX (95 patients) and CAPOX (7 patients). Of the 9 patients who did not receive adjuvant chemotherapy, 1 patient had surgical complications because of which treatment could not be initiated. Of the 93 patients who received adjuvant chemotherapy, 3 patients had treatment discontinuation because of adverse effects and 1 patient, because of recurrence during treatment.

**Table 1:** Clinical characteristics of the patient cohort

	*N* = 102
Sex, N (%)	
Male	69 (67.6)
Female	33 (32.4)
Age (years), median (range)	60 (27–74)
BMI (kg/m^2^), median (range)	23.2 (14.8–33.4)
AV distance (cm), median (range)	4.0 (0.0–8.0)
CEA level (ng/mL), median (range)	3.6 (0.1–381.0)
cT stage N (%)	
T2/T3/T4	2 (2.0)/ 77 (75.5)/ 23 (22.5)
cN stage, N (%)	
Negative	52 (51.0)
Positive	50 (49.0)
Clinical LPLN metastasis, N (%)	
Negative	78 (76.5)
Positive	24 (23.5)
Clinical CRM, N (%)	
Negative	73 (71.6)
Positive	29 (28.4)
mrEMVI, N (%)	
Negative	78 (76.5)
Positive	24 (23.5)
ymrEMVI, N (%)	
Negative	80 (78.4)
Positive	22 (21.6)
Surgical procedure, N (%)	
Low anterior resection	16 (15.6)
Intersphincteric resection	73 (71.6)
Abdominoperineal resection	12 (11.8)
Total colectomy	1 (1.0)
Operation type, N (%)	
Open	33 (32.4)
Laparoscopic	69 (67.6)

*BMI* body mass index, *AV* anal verge, *CEA* carcinoembryonic antigen, *LPLN* lateral pelvic lymph node, *CRM* circumferential resection margin, *mrEMVI* extramural venous invasion based on magnetic resonance imaging before neoadjuvant chemotherapy, *ymrEMVI* extramural venous invasion based on magnetic resonance imaging after neoadjuvant chemotherapy

Forty-seven and 55 patients were classified as good and poor responders, respectively. The median timing of restaging MRI was 2 weeks (range: 0–6 weeks) after the administration of the last round of chemotherapy. The differences between good and poor responders are summarized in Tables 2 and 3. No statistically significant difference was observed in any of the demographic or clinical characteristics. With respect to the pathological and postoperative characteristics, only the ypT stage differed significantly between good and poor responders (*p* = 0.004).

**Table 2:** Comparison of the clinical characteristics of good responders and poor responderss

	Total (*N* = 102)	Good responders (*N* = 47)	Poor responders (*N* = 55)	*p*
Sex, N (%)				
Male	69 (67.6)	28 (59.6)	41 (74.5)	0.11
Female	33 (32.4)	19 (40.4)	14 (25.5)	
Age (years), N (%)				
< 65	80 (78.4)	35 (74.5)	45 (81.8)	0.37
≥ 65	22 (21.6)	12 (25.5)	10 (18.2)	
AV distance (cm), N (%)				
< 5	62 (60.8)	32 (68.1)	30 (54.5)	0.16
≥ 5	40 (39.2)	15 (31.9)	25 (45.5)	
CEA level (ng/mL), N (%)				
< 5	69 (67.6)	30 (63.8)	39 (70.9)	0.45
≥ 5	33 (32.4)	17 (36.2)	16 (29.1)	
cT stage, N (%)				
T2	2 (2.0)	1 (2.1)	1 (1.8)	0.17
T3	77 (75.5)	38 (80.9)	39 (70.9)	
T4	23 (22.5)	8 (17.0)	15 (27.3)	
cN stage, N (%)				
Negative	52 (51.0)	26 (55.3)	26 (47.3)	0.42
Positive	50 (49.0)	21 (44.7)	29 (52.7)	
Clinical LPLN metastasis, N (%)				
Negative	78 (76.5)	37 (78.7)	41 (74.5)	0.62
Positive	24 (23.5)	10 (21.3)	14 (25.5)	
Clinical CRM, N (%)				
Negative	73 (71.6)	34 (72.3)	39 (70.9)	0.87
Positive	29 (28.4)	13 (27.7)	16 (29.1)	
mrEMVI, N (%)				
Negative	78 (76.5)	38 (80.9)	40 (72.7)	0.34
Positive	24 (23.5)	9 (19.1)	15 (27.3)	
ymrEMVI, N (%)				
Negative	80 (78.4)	39 (83.0)	41 (74.5)	0.30
Positive	22 (21.6)	8 (17.0)	14 (25.5)	

*AV* anal verge, *CEA* carcinoembryonic antigen, *LPLN* lateral pelvic lymph node, *CRM* circumferential resection margin, *mrEMVI* extramural venous invasion based on magnetic resonance imaging before neoadjuvant chemotherapy, *ymrEMVI* extramural venous invasion based on magnetic resonance imaging after neoadjuvant chemotherapy

**Table 3:** Comparison of the pathological and postoperative characteristics of good responders and poor responders

	Good responders (*N* = 47)	Poor responders (*N* = 55)	*p*
ypT stage, N (%)			
T0–2	25 (53.2)	14 (25.5)	0.004
T3 and T4	22 (46.8)	41 (74.5)	
ypN stage, N (%)			
Negative	32 (68.1)	32 (58.2)	0.30
Positive	15 (31.9)	23 (41.8)	
Pathological LPLN, N (%)			
Negative	40 (85.1)	43 (78.2)	0.37
Positive	7 (14.9)	12 (21.8)	
Histological type, N (%)			
Well and moderately differentiated	45 (95.7)	51 (92.7)	0.42
Poorly differentiated	2 (4.3)	4 (7.3)	
Pathological CRM, N (%)			
Negative	42 (89.4)	48 (87.3)	0.74
Positive	5 (10.6)	7 (12.7)	
Distal margin, N (%)			
Negative	47 (100.0)	54 (98.2)	0.54
Positive	0 (0.0)	1 (1.8)	
Adjuvant chemotherapy, N (%)			
Absence	3 (6.4)	6 (10.9)	0.33
Presence	44 (93.6)	49 (89.1)	

*LPLN* lateral pelvic lymph node, *CRM* circumferential resection margin

### Prognosis according to response

**Fig. 2:**
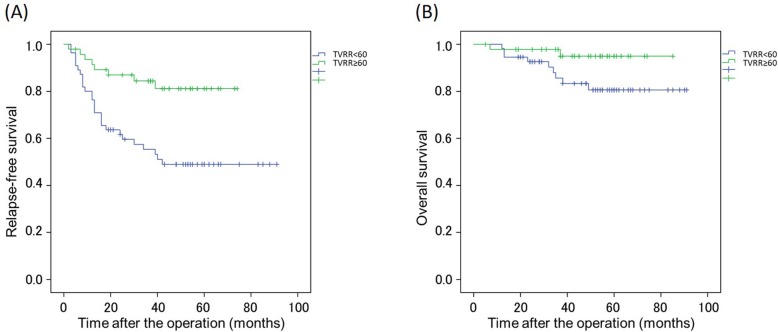
Recurrence-free survival (RFS) and overall survival (OS) of patients with locally advanced low rectal cancer stratified by response to neoadjuvant chemotherapy. **a** Kaplan-Meier curves for RFS in good responders and poor responders (*p* = 0.001). **b** Kaplan-Meier curves for OS in good responders and poor responders (*p* = 0.04). Good and poor response are defined as tumor volume reduction rate (TVRR) of < 60% and ≥ 60%, respectively

The overall 5-year RFS and OS were 63.4 and 87.0%, respectively. Figure 2 shows the Kaplan-Meier curves for RFS and OS in the good and poor responders. The 5-year RFS was 49.0% for poor responders and 81.1% for good responders (*p* = 0.001). The 5-year OS was 80.6% for poor responders and 94.9% for good responders (*p* = 0.06).

### Independent prognostic factors obtainable before surgery

Table 4 shows the results of the univariate and multivariate Cox proportional hazards analyses. In the univariate analyses of RFS, cT stage (*p* = 0.001), clinical CRM (*p* = 0.001), ymrEMVI (*p* < 0.001), and TVRR (*p* = 0.003) were significant prognostic factors. In the multivariate analysis of RFS, ymrEMVI (HR: 2.74, 95% CI: 1.36–5.50, *p* = 0.005) and TVRR (HR: 3.48, 95% CI: 1.57–5.50 *p* = 0.002) were significant independent prognostic factors. In the univariate analyses of OS, clinical CRM (*p* = 0.02) and ymrEMVI (*p* = 0.01) were significant prognostic factors. There was no significant independent prognostic factor for OS.

**Table 4:** Univariate and multivariate analyses of preoperative prognostic factors for recurrence-free survival and overall survival

	Recurrence-free survival						Overall survival					
	Univariate analysis			Multivariate analysis			Univariate analysis			Multivariate analysis		
	HR	95% CI	*p*	HR	95% CI	*p*	HR	95% CI	*p*	HR	95% CI	*p*
Sex												
Male	1.70	0.77–3.74	0.19				1.23	0.33–4.64	0.76			
Female	1						1					
Age (years)												
< 65	1						1					
≥ 65	0.95	0.42–2.18	0.91				1.46	0.39–5.49	0.58			
AV distance (cm)												
< 5	1.29	0.64–2.58	0.48				1.69	0.45–6.39	0.44			
≥ 5	1						1					
CEA level (ng/mL)												
< 5	1						1					
≥ 5	1.80	0.93–3.53	0.84				1.16	0.34–3.97	0.81			
cT stage												
T2 and T3	1			1			1					
T4	3.00	1.53–5.91	0.001	2.01	0.92–4.42	0.08	3.24	0.99–10.62	0.05			
cN stage												
Negative	1						1					
Positive	1.35	0.69–2.62	0.38				0.87	0.27–2.85	0.82			
Clinical LPLN metastasis												
Negative	1						1					
Positive	1.71	0.84–3.48	0.14				1.38	0.37–5.21	0.64			
Clinical CRM												
Negative	1			1			1			1		
Positive	2.99	1.54–5.80	0.001	1.97	0.90–4.35	0.09	4.43	1.30–15.14	0.02	3.24	0.90–11.68	0.07
ymrEMVI												
Negative	1			1			1			1		
Positive	3.37	1.72–6.61	< 0.001	2.74	1.36–5.50	0.005	4.43	1.35–14.51	0.01	3.15	0.91–10.89	0.07
TVRR (%)												
< 60	3.37	1.53–7.42	0.003	3.48	1.57–7.72	0.002	3.90	0.84–18.06	0.08			
≥ 60	1			1			1					

*HR* hazard ratio, *CI* confidence interval, *AV* anal verge, *CEA* carcinoembryonic antigen, *LPLN* lateral pelvic lymph node, *CRM* circumferential resection margin; *ymrEMVI* extramural venous invasion based on magnetic resonance imaging after neoadjuvant chemotherapy, *TVRR* tumor volume reduction rate

**Fig. 3:**
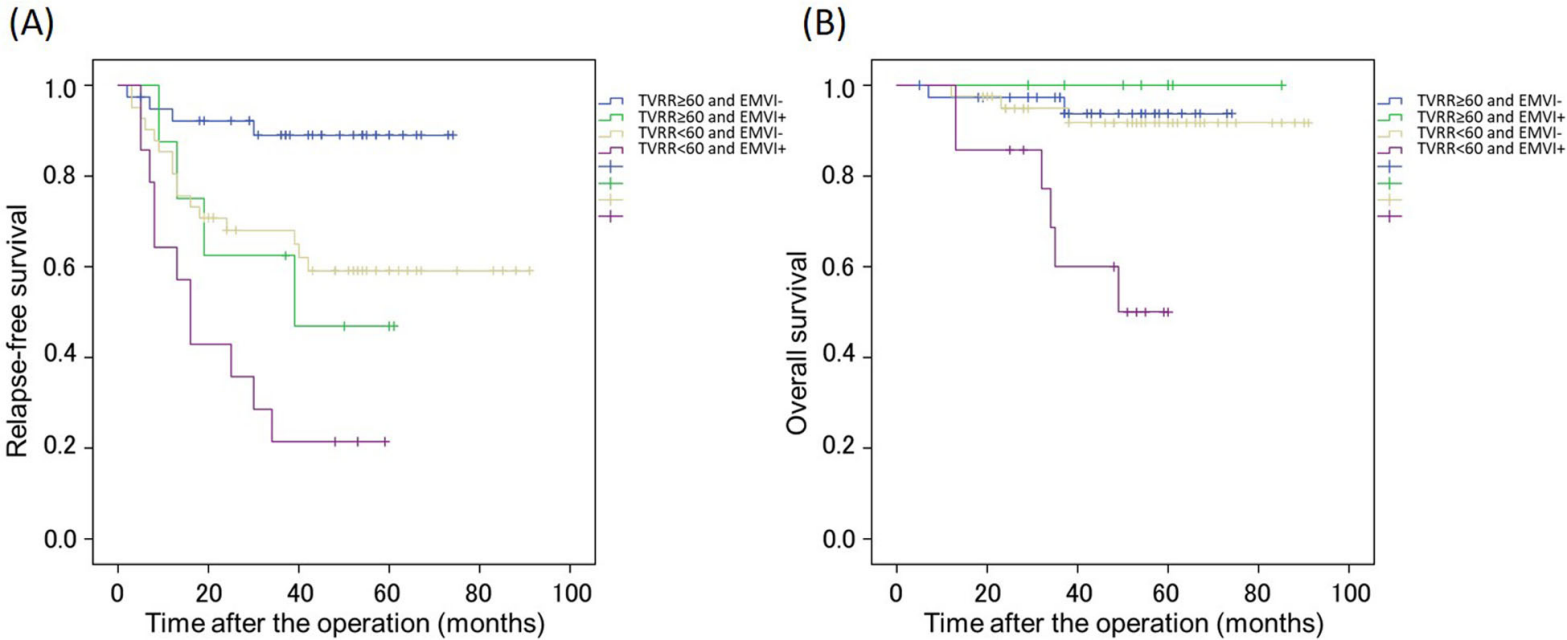
Recurrence-free survival (RFS) and overall survival (OS) in patients with locally advanced low rectal cancer stratified by response to neoadjuvant chemotherapy (good vs. poor) and the presence of extramural venous invasion on magnetic resonance imaging (EMVI; positive vs. negative). **a** Kaplan-Meier curves for RFS in the response and EMVI strata (*p* < 0.001). **b** Kaplan-Meier curves for OS in the response and EMVI strata (*p* = 0.002). Good and poor response are defined as tumor volume reduction rate (TVRR) of < 60% and ≥ 60%, respectively

Figure 3 shows the Kaplan-Meier curves for RFS (*p* < 0.001) and OS (*p* < 0.001) stratified by response to NAC and ymrEMVI status. Good responders without ymrEMVI had the best 5-year RFS and OS (89.0 and 93.8%, respectively). Poor responders with ymrEMVI had the worst 5-year RFS and OS (21.4 and 50.0%, respectively). Good responders with ymrEMVI (5-year RFS, 46.9%; 5-year OS, 91.8%) and poor responders without ymrEMVI (5-year RFS, 59.1%; 5-year OS, 100.0%) had similar survival outcomes.

### Recurrence pattern and rate according to response and EMVI

Thirty-five patients (34.3%) experienced recurrence; 20 patients (19.6%) had local recurrence; 21 patients (20.6%) had distant metastasis, including 10 (9.8%) lung metastasis, 9 (8.8%) distant lymph node metastasis, and 3 (2.9%) liver metastasis. The 5-year local recurrence rate (LRR) was 34.0% for poor responders and 11.5% for good responders (*p* = 0.02). The 5-year distant metastasis-free survival (DFS) was 64.4% for poor responders and 91.7% for good responders (*p* = 0.001). The 5-year LRR was 16.5% for negative ymrEMVI and 48.2% for positive ymrEMVI (*p* = 0.002). The 5-year DFS was 83.7% for negative ymrEMVI and 49.7% for positive ymrEMVI (*p* = 0.004).

## Discussion

We found that tumor volume reduction after NAC was associated with a better prognosis in patients with LALRC, even in patients with features that suggest a very high risk of recurrence. Patients with good responses to NAC had better outcomes (5-year RFS, 81.1%; 5-year OS, 94.9%) than patients with poor responses to NAC (5-year RFS, 49.0%; 5-year OS, 80.6%). The best responders had better survival outcomes than the poorest responders. Particularly, all 7 patients with TVRR > 90% experienced cancer-free survival with only 1 patient of resectable lung recurrence. However, of the 7 patients with TVRR < 0%, 6 patients developed unresectable recurrences. Extreme response to NAC could be a useful factor for predicting prognosis and selecting individualized treatment strategies. Additionally, we found that preoperative EMVI was associated with a worse prognosis. In poor responders with ymrEMVI, the 5-year RFS and OS were 21.4 and 50.0%, respectively. However, in good responders without ymrEMVI, the 5-year RFS and OS were 89.0 and 93.8%, respectively. TVRR and EMVI were associated with significantly poorer survival outcomes including local and distant recurrence, even when TME and LPLND were performed after NAC.

Survival outcomes can be predicted from tumor volume reductions after preoperative CRT [13, 16, 17]. MRI-based assessments of response to CRT are associated with survival outcomes, including RFS and OS. Response to preoperative chemotherapy is reportedly associated with tumor regression grade and downstaging; however, associations with prognosis have not been reported [18]. Our study revealed that the TVRR was associated with RFS and OS, and that response to preoperative chemotherapy was predictive of survival, consistent with the findings of prior studies on CRT. Poor response to NAC was associated with worse survival outcomes. There are several potential reasons for this finding, including the biological malignancy of the tumor, sensitivity to the administered drugs, and host immunity. Very good responders may not need NACRT to achieve local disease control. Several randomized controlled trials are currently investigating this topic. Conversely, it may be difficult for very poor responders to obtain good prognosis even when they receive multidisciplinary treatments, such as total neoadjuvant therapy.

EMVI, including mrEMVI and ymrEMVI, was a significant preoperative prognostic factor in this study. EMVI is defined as the presence of tumor cells within blood vessels beyond the extramural area near the primary tumor. The incidence of EMVI in LALRC is approximately 9–61% [19]. Histological EMVI is a poor prognostic factor [20, 21], and mrEMVI is an independent risk factor for poor survival outcomes [5, 22, 23]. Therefore, the European Society for Medical Oncology guidelines suggest that mrEMVI indicates high risk; CRT followed by TME is recommended in such cases [24]. Moreover, several studies have shown that the presence of ymrEMVI, which was evaluated after the neoadjuvant treatment, and not only mrEMVI, had prognostic impact [23, 25–27]. Our study revealed that mrEMVI remained after NAC except for 2 patients. EMVI hardly disappears after NAC and may not lead to an improvement in prognosis, even if NAC is performed. EMVI was associated with poor survival outcomes, even in good responders. Indeed, the survival outcomes of good responders with EMVI were similar to those of poor responders. Our results suggest that EMVI is a strong prognostic factor for LALRC. Therefore, the presence of EMVI might require separate treatment strategies.

The local recurrence rate for the whole cohort was high (19%). We selected NAC followed by surgery for patients with high-risk features for distant metastasis, which was regarded as the key factor for survival. In the end, our strategy caused a high rate of local recurrence that might have had a negative effect on patient survival. Further, the results show that NACRT is essential for local control even in patients at high risk of distant recurrence, if the aim is to cure the disease. Good response to NAC without EMVI was strongly associated with good prognosis. Patients who showed very good responses to NAC did not necessarily require NACRT. However, lymph node metastasis, EMVI, and very low location of the tumor were high-risk features for both local and distant recurrences; therefore, not only NAC but also NACRT should be considered for disease control. In our facilities, there has been a tendency to omit radiation therapy and to choose anal-sparing surgery. As the number of patients who undergo NACRT increases, there will be reductions in the rate of anal-sparing surgery, which is strongly affected by CRT in terms of anal function. Although ISR cannot be expected to increase the CRM-positive rate for the surrounding organs and neural tissues, except for the levator ani muscles, the rate of anal-sparing surgery might also affect the rate of local recurrence.

The present study had several limitations. First, it used a retrospective, single-institution study design, and had a small sample size. Several prospective trials of preoperative chemotherapy for LALRC are ongoing, and their results are expected to lead to tailored treatments. Second, the patients received different NAC regimens, such as FOLFOX and CAPOX. However, these regimens were considered to have almost the same therapeutic outcome [28]. Therefore, the differences between these regimens would not have had a substantial effect on the prognosis in each group. Third, the methods of evaluating tumor volume reduction, mrEMVI, and ymrEMVI might not be universally applicable. Moreover, in this study, the size of primary lesions and existence of EMVI were evaluated by two independent experienced colorectal surgeons. Unlike Western radiologists, many Japanese radiologists are unfamiliar with rectal MRI, including EMVI. Therefore, although independent radiologists should have reviewed these factors, two independent experienced colorectal surgeons reviewed the factors, and incongruent results were reviewed and finalized by consensus. However, despite these limitations, our results suggest that these factors after NAC were important prognostic factors for patients with LALRC and might be used to identify patients who will have a good or poor prognosis.

## Conclusion

The findings of this retrospective study suggest that tumor volume reduction after NAC is associated with the prognosis of LALRC. Patients with good responses to NAC have better survival outcomes. Further, TVRR and EMVI are significant and independent preoperative prognostic factors for RFS, including local and distant recurrence. EMVI was also associated with significantly worse survival outcomes in stratified analyses.

## Data Availability

Not applicable.

## Abbreviations

95% CIs: 95% confidence interval
AV: Anal verge
CAPOX: Capecitabine and oxaliplatin
CRM: Circumferential resection margin
CT: Computed tomography
DFS: Distant metastasis-free survival
FOLFOX: Folinic acid, fluorouracil, and oxaliplatin
HR: Hazard ratio
ISR: Intersphincteric resection
LALRC: Locally advanced lower rectal cancer
LPLND: Lateral pelvic lymph node dissection
LRR: Local recurrence rate
mrEMVI: Extramural venous invasion based on magnetic resonance imaging before neoadjuvant chemotherapy
MRI: Magnetic resonance imaging
NAC: Neoadjuvant chemotherapy
NACRT: Neoadjuvant chemoradiotherapy
OS: Overall survival
RFS: Recurrence-free survival
TME: Total mesorectal excision
TVRR: Tumor volume reduction rate
ymrEMVI: Extramural venous invasion based on magnetic resonance imaging after neoadjuvant chemotherapy
